# When vertically aligned carbon nanotube microstructures lose verticality: experimental and computational insights into geometry-dependent stability

**DOI:** 10.1039/d6ra07679c

**Published:** 2026-09-15

**Authors:** Ozlem Simsek, O. Tolga Gul

**Affiliations:** a Physics Department, Polatlı Faculty of Science and Letters, Ankara Hacı Bayram Veli University Ankara 06900 Türkiye tolga.gul@hbv.edu.tr

## Abstract

Patterned vertically aligned carbon nanotube (VACNT) microstructures are promising building blocks for three-dimensional carbon architectures, yet the geometric conditions governing preservation of their as-grown verticality remain poorly resolved. Here, we systematically map the loss of verticality of square VACNT microstructures over 16 combinations of catalyst footprint and growth condition, spanning lateral dimensions of 12.5–75 µm and footprint-specific mean heights of approximately 24–231 µm. As-grown deformation was quantified from scanning electron microscopy images using a dimensionless projected relative displacement metric, *D*. Although deformation generally increased as the structures became more slender, the response did not follow a universal *H̄*/*L* scaling. Instead, distinct low, transition, and advanced-deformation regimes emerged, and geometries with nearly identical *H̄*/*L* ≈ 3 exhibited almost a sevenfold difference in median *D*. This breakdown of aspect-ratio-only scaling demonstrates that absolute footprint and height, and the growth-dependent evolution associated with them, remain important in determining the final morphology. Orthotropic finite element eigenvalue analysis nevertheless revealed a systematic reduction in critical pressure with increasing *H̄*/*L* and converged toward the shear-corrected Timoshenko response in the beam-column regime. The resulting susceptibility index showed a moderate positive association with experimental deformation (*ρ*_s_ = 0.58, *n* = 12), indicating that elastic instability provides an important geometric contribution but does not uniquely determine the as-grown state. These findings distinguish geometric susceptibility from final morphological evolution and provide a quantitative framework for designing patterned VACNT architectures that retain verticality.

## Abstract 2

Loss of verticality in patterned VACNT microstructures depends on both footprint and height. Experiments and FE analysis reveal a transition footprint and show that the response cannot be described by a universal *H*/*L* scaling alone.

## Graphical abstract

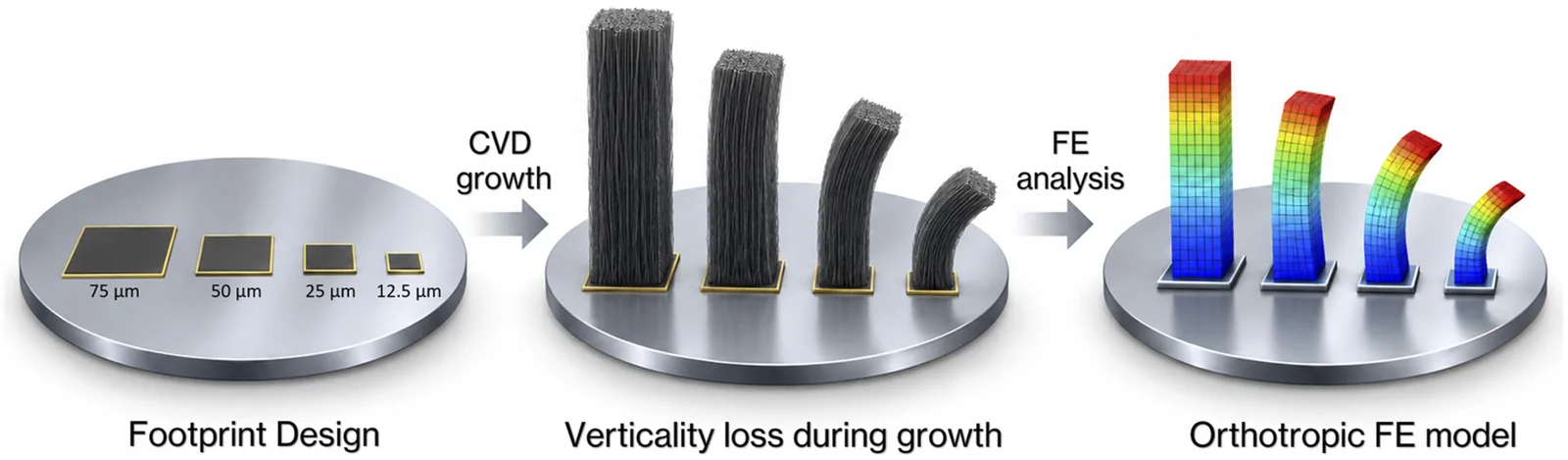

## 1 Introduction

Vertically aligned carbon nanotube (VACNT) forests offer a bottom-up route for fabricating porous, anisotropic, and high aspect ratio structures whose dimensions extend from the nanotube scale to macroscopic heights.^1–3^ Advances in chemical vapor deposition (CVD) have enabled the rapid synthesis of millimeter-scale VACNT films as well as lithographically defined microstructures from patterned thin film catalysts.^1–3^ When the catalyst is patterned, the footprint, spacing, and arrangement of VACNT structures can be prescribed independently of their nanoscale forest morphology.^3–6^ This capability has supported the development of three-dimensional microelectromechanical devices, interconnect-like structures, functional interfaces, and surfaces with tailored wetting behavior.^4,5,7–12^ Their technological value arises not only from the intrinsic electrical, thermal, and mechanical properties of VACNTs, but also from the collective architecture and orientation of the forest.^5,8–10,13,14^ However, the performance and manufacturability of a patterned VACNT structure depend critically on whether the intended vertical geometry is retained during growth.^3,6,13,15^ A tilted, curved, or laterally displaced structure can alter its contact area, spacing, directional response, and compatibility with subsequent integration or coating processes.^4,6–10^ Such deviations become particularly important when the footprint is reduced or the structure height is increased, because relatively small lateral perturbations can generate substantial angular and positional errors.^6^ Understanding how the lateral footprint and height govern the as-grown verticality of patterned VACNT microstructures is therefore essential for geometry-controlled manufacturing and applications requiring repeatable three-dimensional architectures.^4–7^

VACNT growth is inherently collective rather than a simple superposition of independently elongating nanotubes.^13,16–19^ More recent studies have further emphasized the link between CNT forest growth, morphology, and geometric uniformity, including predictive structure–property approaches and process strategies aimed at suppressing spatial nonuniformity during CVD.^20,21^ The crowding effect among neighboring VACNTs can transform an initially disordered population into a highly aligned forest, demonstrating that lateral confinement and mechanical interaction play central roles in vertical organization.^13^ Growing VACNT films can also generate measurable forces, while externally applied constraints can influence their height, alignment, and final microscale shape.^22^ Jeong *et al.* showed that catalyst pattern dimensions and inter-pattern spacing affect both the height and vertical alignment of VACNT pillar arrays, directly linking lithographic geometry to the resulting morphology.^6^ Collective growth models have further related forest evolution and self-termination to mechanical contact, variations in nanotube growth activity, and the loss of a self-supporting population structure.^16,17,19^ Population-level measurements have revealed substantial spatial and temporal heterogeneity in CNT nucleation, elongation, density, and catalyst activity even under nominally uniform processing conditions.^17–19,23^ For patterned arrays, chemically mediated coupling between neighboring catalyst regions can additionally produce size and spacing-dependent differences in micropillar growth rate and uniformity.^24^ Real-time environmental transmission electron microscopy has confirmed that forest formation involves simultaneous self-organization and mechanical competition among VACNTs having heterogeneous nucleation times and growth rates.^25^ These interactions have also been deliberately exploited through differential growth patterning to manufacture uniformly curved and freeform CNT microstructures.^26^ These studies establish that VACNT shape emerges from coupled reaction kinetics, transport, crowding, and mechanical interaction, but they have mainly addressed height uniformity, nanoscale alignment, growth termination, or intentionally programmed curvature rather than unintended geometry-dependent loss of verticality.

A complementary body of research has examined the mechanical response of VACNT forests and micropillars after growth, particularly under externally imposed axial compression.^27–40^ These studies have reported foam-like deformation, recoverability, strain localization, and coordinated buckling arising from the collective response of large CNT populations. Cao *et al.* observed the initiation and propagation of localized buckling regions during compression, demonstrating that structural instability can dominate deformation well before complete material collapse.^33^ Pathak *et al.* showed that local relative density gradients strongly influence the strength, deformation sequence, and failure behavior of VACNT structures.^37^ Maschmann *et al.* subsequently demonstrated that transversely isotropic continuum descriptions, together with Timoshenko-type stability analysis, can capture the geometry-dependent critical buckling stress of patterned VACNT columns.^38^ More recent studies of architected VACNT foams have further demonstrated that mesoscale geometry, geometric confinement, and hierarchical interactions can modify deformation modes and mechanical property scaling, reinforcing the importance of geometry in the collective structure-level response.^41–44^ These findings support treating a patterned VACNT microstructure as an effective anisotropic solid when the objective is to assess structure level stability rather than individual nanotube deformation. Nevertheless, most mechanical investigations have focused on continuous forests or micropillars subjected to post growth compression and have primarily examined stress–strain response, localized buckle formation, recoverability, or energy dissipation. Conversely, growth-oriented studies have generally quantified height, growth kinetics, catalyst coupling, and alignment without systematically relating the as-grown lateral displacement of individual structures to both footprint and height.^6,13,16–19,22–26^ Accordingly, a quantitative framework that systematically spans both footprint and height and directly compares experimentally observed loss of verticality with a continuum stability metric remains limited.^6,37,38^

Here, we address the geometry-dependent loss of verticality in lithographically patterned VACNT microstructures by examining how catalyst footprint size and growth-dependent structure height influence their as-grown morphology. Quantitative SEM analysis is combined with continuum stability modeling to determine whether geometric slenderness alone can account for the observed deformation, or whether absolute dimensions and growth-dependent evolution must also be considered. By focusing on spontaneous as-grown displacement rather than deliberately programmed curvature or post-growth compression, this study establishes a framework for identifying geometries that are more likely to retain their intended vertical architecture.

## 2 Experimental

### 2.1 Catalyst patterning and VACNT growth

**Fig. 1:**
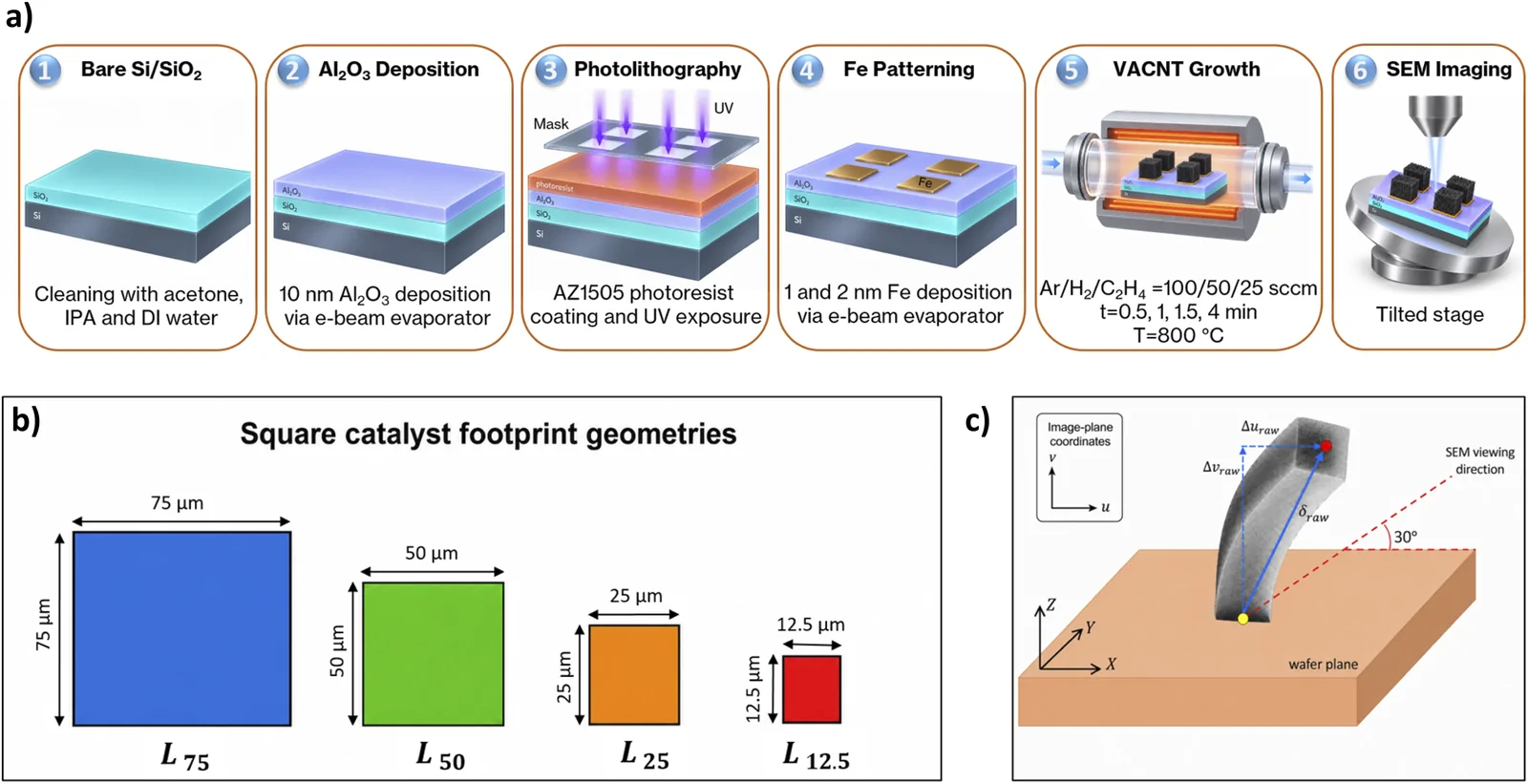
Experimental workflow, geometric design, and image-based measurement of projected VACNT microstructure deformation. (a) Schematic illustration of the experimental procedure, including substrate preparation, Al_2_O_3_ deposition, photolithographic patterning, Fe catalyst deposition, VACNT growth by CVD, and inclined SEM imaging. (b) Top view schematics of the square catalyst footprints used in this study: *L*_75_, *L*_50_, *L*_25_, and *L*_12.5_, corresponding to side lengths of 75, 50, 25, and 12.5 µm, respectively. (c) Schematic representation of the inclined SEM imaging geometry and image-plane displacement measurement.

The overall experimental workflow, from substrate preparation and catalyst patterning to VACNT growth and SEM imaging, is summarized schematically in Fig. 1a. First, square catalyst patterns were fabricated on Si substrates covered with a 300 nm thermally grown SiO_2_ layer. Before lithographic processing, the substrates were cleaned using acetone, IPA and DI water and dried with N_2_. A 10 nm Al_2_O_3_ catalyst-support and diffusion barrier layer was deposited by e-beam evaporation at a deposition rate of 0.5 nm s^−1^. AZ1505 photoresist was subsequently spin-coated at 4000 rpm for 1 min and soft-baked at 60 °C for 1.5 min. The square catalyst footprints were defined by UV photolithography using a mask aligner. Following exposure, the patterns were developed in AZ400K for 45 s, rinsed with DI water, and dried under N_2_.

The patterned catalyst layout contained square footprints with side lengths of 75, 50, 25, and 12.5 µm, corresponding to footprint areas of 75 × 75, 50 × 50, 25 × 25, and 12.5 × 12.5 µm^2^, respectively. Fe catalyst layers with nominal thicknesses of 1 and 2 nm were deposited by e-beam evaporation at a deposition rate of 0.25 nm s^−1^. The 1 and 2 nm Fe catalyst regions were produced using two separate lithographic/deposition sequences. After deposition, lift-off was performed in acetone for 1 min, followed by rinsing with DI water and drying under N_2_. The 1 nm Fe catalyst patterns were used for all quantitative height, displacement, and verticality analyses. The adjacent 2 nm Fe catalyst rows were included on the same substrates to provide a qualitative visual comparison of the catalyst thickness-dependent VACNT height contrast in the SEM images, but they were excluded from all quantitative measurements and statistical analyses.

VACNT growth was carried out by atmospheric pressure CVD in a horizontal quartz tube furnace. The patterned substrates were positioned at a fixed location within the heated zone, with the same orientation relative to the gas-flow direction. Before heating, the reactor was purged with Ar at 400 sccm for 10 min. The furnace was then heated to 800 °C at a rate of 100 °C min^−1^ under a flow of Ar. After the growth temperature was reached, the Fe catalyst was annealed and reduced for 5 min under Ar and H_2_ flow rates of 100 and 75 sccm, respectively. VACNT growth was initiated by introducing C_2_H_4_, resulting in growth-stage flow rates of 100, 50, and 25 sccm for Ar, H_2_, and C_2_H_4_, respectively.^45,46^ Growth durations of 0.5, 1.0, 1.5, and 4.0 min, hereafter denoted as *t*_0.5_, *t*_1.0_, *t*_1.5_, and *t*_4.0_, respectively, produced footprint-specific mean VACNT heights of approximately 25, 55, 75, and 225 µm. At the end of each growth period, the C_2_H_4_ flow was terminated, and the reactor was cooled under Ar at a flow rate of 300 sccm. The samples were removed after the furnace temperature decreased below 50 °C. Three independent growth experiments were performed for each growth-time condition. In each experiment, all four footprint sizes were incorporated on the same chip and therefore experienced the same nominal growth conditions. The footprint-specific mean heights measured for each growth-time condition are reported in Table 1. The resulting VACNT microstructures were characterized using a scanning electron microscope (SEM) operated at an accelerating voltage of 5.0 kV and a working distance of 9–10 mm. SEM images used for the morphological and projected verticality analyses were acquired using an inclined imaging geometry, in which the viewing direction was approximately 30° relative to the wafer plane.

**Table 1:** Footprint-specific mean heights and corresponding aspect ratios obtained at different growth times. Heights are reported as the mean of three independent chip-level means ± standard deviation (*n* = 3 independent growth experiments). Within each chip, approximately 11, 13, 26, and 47 structures were measured for *L*_75_, *L*_50_, *L*_25_, and *L*_12.5_, respectively. Aspect ratios were calculated using the corresponding footprint-specific mean heights

Growth time (min)	*H̄*_*t*,75_ (µm)	*H̄*_*t*,75_/*L*_75_ (-)	*H̄*_*t*,50_ (µm)	*H̄*_*t*,50_/*L*_50_ (-)	*H̄*_*t*,25_ (µm)	*H̄*_*t*,25_/*L*_25_ (-)	*H̄*_*t*,12.5_ (µm)	*H̄*_*t*,12.5_/*L*_12.5_ (-)
0.5	23.7 ± 1.2	0.316	24.3 ± 0.6	0.487	25.7 ± 1.5	1.027	25.3 ± 0.6	2.027
1.0	53.7 ± 1.5	0.716	53.7 ± 2.1	1.073	55.7 ± 0.6	2.227	58.0 ± 1.0	4.640
1.5	71.3 ± 0.6	0.951	73.3 ± 1.5	1.467	76.7 ± 1.5	3.067	78.7 ± 1.2	6.293
4.0	222.3 ± 2.5	2.964	221.7 ± 3.2	4.433	228.3 ± 2.5	9.133	231.0 ± 2.0	18.480

### 2.2 Geometric design and image-based quantification of VACNT microstructure verticality

Square catalyst patterns with lateral dimensions of 75 × 75 µm^2^, 50 × 50 µm^2^, 25 × 25 µm^2^, and 12.5 × 12.5 µm^2^ were used to investigate the influence of lateral footprint size on the vertical stability of VACNT microstructures. Throughout the manuscript, these geometries are denoted as *L*_75_, *L*_50_, *L*_25_, and *L*_12.5_, respectively, where *L* represents the side length of the square catalyst footprint (Fig. 1b). All structures included in the quantitative analysis were grown from 1 nm Fe catalyst patterns. Four growth-time conditions *t*_0.5_, *t*_1.0_, *t*_1.5_, and *t*_4.0_, were investigated. Because the measured VACNT height varied slightly with footprint size at a given growth time, the footprint-specific mean height is denoted as *H̄*_*t*,*L*_, and the corresponding aspect ratio as *H̄*_*t*,*L*_/*L*. The complete geometric matrix is summarized in Table 1.

SEM images were acquired at an observation angle of approximately 30° relative to the wafer plane, corresponding to approximately 60° relative to the nominal surface normal direction. A sample-fixed coordinate system was defined such that *X* and *Y* lie within the wafer plane, while *Z* represents the surface normal and nominal VACNT growth direction (Fig. 1c). Because a single SEM image provides only a two-dimensional projection, displacement was quantified in the image-plane coordinate system (*u*,*v*), where *u* and *v* correspond to the horizontal and vertical image directions, respectively. Thus, all reported deformation quantities represent projected image-plane displacements rather than the full three-dimensional displacement.

For each structure *i*, the basal position and upper VACNT position were defined as the approximate geometric centers of the visible basal footprint and upper face, respectively, and were manually identified in Fiji/ImageJ. No automated centroid-detection macro was used. The raw image-plane displacement components were defined as Δ*u*_raw,*i*_ and Δ*v*_raw,*i*_, giving
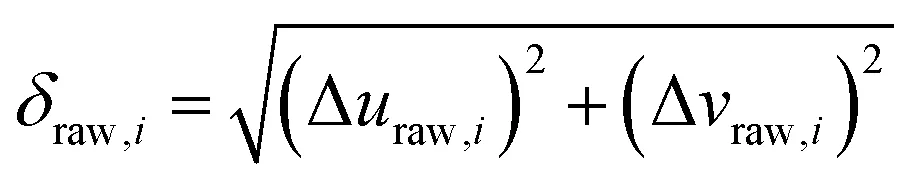


Coordinates were obtained using a custom Fiji/ImageJ workflow. For each footprint-size row, the basal and upper-face centers were identified using multipoint selections. The basal centers were fitted by principal component analysis to determine the local row direction and correct for small in-plane rotations of the patterned rows. Basal and upper-face points were then ordered along this direction and paired from left to right (Fig. 1c).

Because the SEM images were acquired at an inclined viewing geometry, even an ideally vertical structure produces a finite apparent base to top displacement in the image plane. Using the angular convention shown in Fig. 1c, a vertical structure of height *H̄*_*t*,*L*_ produces a projected displacement magnitude of*δ*_proj_ = *H̄*_*t*,*L*_ cos 30° ≈ 0.866*H̄*_*t*,*L*_

The raw displacement components were therefore normalized by the corresponding footprint and growth time-specific mean height, *H̄*_*t*,*L*_, before reference subtraction. For an ideally vertical structure, the magnitude of the normalized projection contribution is consequently
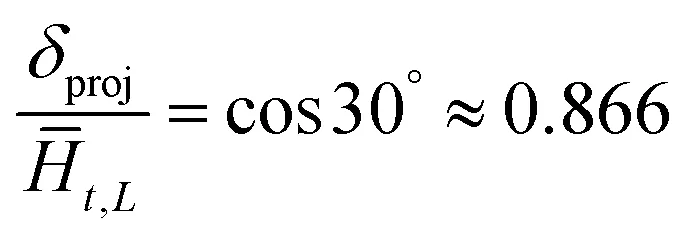
which is independent of the absolute mean structure height. Differences in *H̄*_*t*,*L*_ among the footprint groups therefore do not, by themselves, introduce a systematic height-dependent contribution to the normalized displacement. Variations in the heights of individual structures around the corresponding *H̄*_*t*,*L*_ may contribute to within-group scatter, but do not produce the systematic dependence on mean height that would arise from using the unnormalized projected displacement.

The normalized image-plane displacement vector for structure *i* was defined as
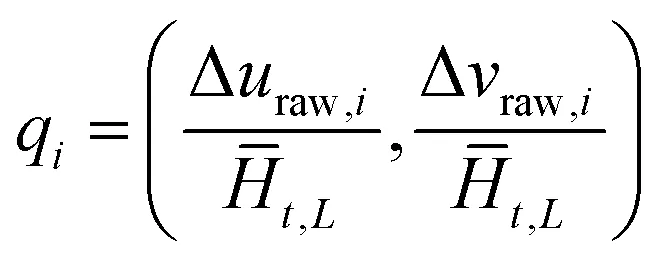


To remove the common normalized projection contribution and image-specific offsets, the *L*_75_ population within each SEM image was used as an internal reference. *L*_75_ was selected because it represents the largest footprint and the lowest geometric slenderness at each growth condition and consistently retained the most columnar morphology in the SEM observations. Importantly, this choice does not imply that the *L*_75_ structures are physically undeformed or perfectly vertical. Rather, the component-wise median normalized displacement vector of the *L*_75_ population provides an image-specific reference. The corrected displacement vector was therefore calculated as
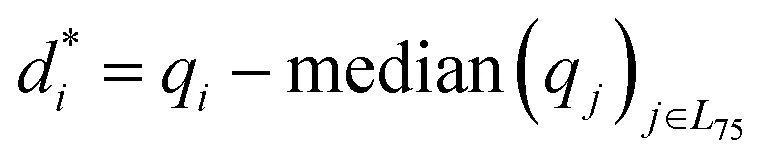
where the median was evaluated component-wise over the *L*_75_ population within the same SEM image. The magnitude of the corrected vector was then used as the projected relative displacement metric,
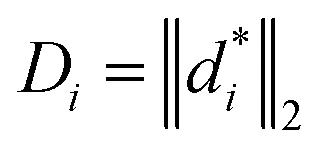


Accordingly, *D*_*i*_ quantifies the projected deformation of an individual VACNT microstructure relative to the median *L*_75_ response within the same SEM image and should not be interpreted as an absolute three-dimensional inclination. The height normalization removes the systematic dependence of the common projection contribution on the footprint-specific mean height, while the *L*_75_-based correction removes the remaining common image-plane reference contribution. However, because only a single inclined SEM projection was available, out of plane deformation cannot be uniquely reconstructed, and residual variation associated with individual heights may contribute to the within-group scatter. *D*_*i*_ is therefore intentionally interpreted as a projected relative displacement metric.

To assess synthesis to synthesis reproducibility, the projected displacement analysis was performed independently for three chips at each growth time condition. For each chip, the normalization and *L*_75_-based reference correction were applied independently using the corresponding footprint-specific mean heights and displacement data from that chip. Individual VACNT structures within a chip were treated as structural subsamples, whereas the independently grown chips were treated as synthesis replicates. The detailed individual structure distributions presented in Fig. 3 correspond to one representative chip for each growth time condition, while reproducibility across the three independent growth experiments was evaluated using chip-level median *D* values (Fig. S1).

### 2.3 Finite element modeling and eigenvalue-based stability analysis

Finite element analyses were performed in Abaqus/Standard to assess the geometry-dependent mechanical stability of the VACNT microstructures. Each structure was idealized as a homogeneous orthotropic column with a square cross section of *L* × *L* and a footprint-specific mean height *H̄*_*t*,*L*_, corresponding to the 16 experimental geometries listed in Table 1. A literature informed idealized orthotropic property set was adopted. The longitudinal modulus was set to *E*_3_ = 297 MPa, which lies within the reported effective compressive modulus range of 100–300 MPa for patterned VACNT turfs and is close to the experimentally measured axial modulus range of 165–275 MPa for transversely isotropic CNT forests.^38,47^ The transverse and shear properties were assigned through the ratios *E*_1_/*E*_3_ = *E*_2_/*E*_3_ = *G*_13_/*E*_3_ = *G*_23_/*E*_3_ = 0.10 and *G*_12_/*E*_3_ = 0.05 (Table S1). All Poisson's ratios were set to zero, and the longitudinal material direction, *E*_3_, was aligned with the nominal VACNT growth direction, *Z*. These effective properties were used as a common orthotropic baseline for comparing all 16 geometries. Therefore, FE results are interpreted primarily in terms of relative geometric trends.

The bottom surface of each model was fully constrained to represent attachment to the substrate. A reference point was positioned at the center of the upper surface and connected to the surface through a uniformly weighted distributing coupling. Only the axial translational component, *U*_3_, was included in the coupling. The compressive reference load was applied in the negative *Z* direction as an equivalent resultant force,*F*_ref_ = *p*_ref_*L*^2^where *p*_ref_ = 1 MPa. Because the lateral translations and rotations of the upper surface were not coupled to the reference point, the loading arrangement represented a fixed base with a laterally and rotationally free loaded end. The models were discretized using eight-node linear brick elements with incompatible modes (C3D8I). A 6 × 6 cross-sectional mesh was used for the complete geometry matrix. Mesh convergence was evaluated for two representative geometries with *H̄*_*t*,*L*_/*L* = 3.067 and 18.480 using 4 × 4, 6 × 6, and 8 × 8 cross-sectional discretizations. The 6 × 6 mesh differed from the corresponding 8 × 8 result by 0.298% and 0.0056%, respectively, and was therefore adopted for all subsequent analyses (Table S2).

Linear perturbation eigenvalue-buckling analyses were conducted using the subspace eigensolver, with eight eigenmodes requested for each geometry. The first positive eigenvalue, *λ*_1_, was used to calculate the FE critical pressure as*p*_cr,FE_ = *λ*_1_*p*_ref_

Because the modeled cross sections were square, the first two eigenvalues were generally identical or nearly identical and represented orthogonal lateral bending modes. For visualization, each eigenmode displacement field was normalized independently by its maximum value; therefore, the displayed contours represent only the spatial mode shape and not the physical displacement amplitude at instability.

The FE critical pressures were compared with a shear-corrected Timoshenko formulation for a fixed-free square column using a shear correction factor of 5/6. This comparison was interpreted primarily for geometries with *H̄*/*L* ≥ 1, while structures with *H̄*/*L* < 1 were treated as non-slender configurations outside the classical beam-column regime. To relate the numerical stability results to the experimental deformation measurements, an FE-based susceptibility index, *Π*_FE_, was defined by normalizing the critical pressure relative to the *L*_25_–*t*_1.5_ condition. The association between *Π*_FE_ and the experimental median *D* values was evaluated using Spearman's rank correlation coefficient for the 12 geometries satisfying *H̄*/*L* ≥ 1. The complete experimental, numerical, analytical, and susceptibility index data are provided in Table S3.

Material parameter sensitivity was assessed for four representative geometries spanning *H̄*/*L* ≈ 1, 3, 9, and 18. The longitudinal modulus was maintained at *E*_3_ = 297 MPa, while the transverse and shear moduli were scaled by factors of 0.5 and 2 relative to the baseline set, producing *E*_3_/*G*_13_ ratios of 20, 10, and 5, respectively. This analysis was used to evaluate the robustness of the predicted critical pressure trends to the assumed orthotropic stiffness contrast.

## 3 Results and discussion

### 3.1 Growth time and footprint-dependent morphological evolution

**Fig. 2:**
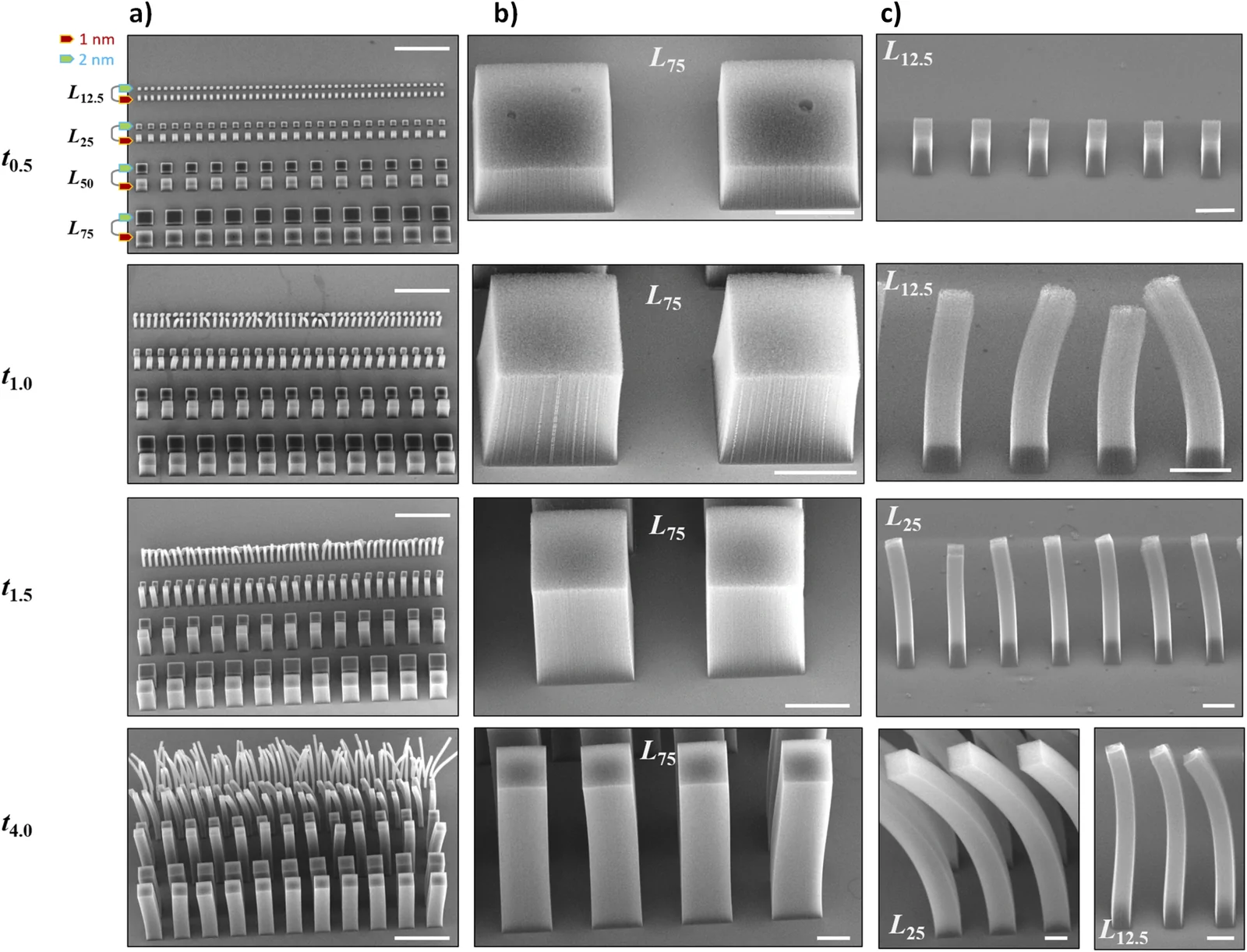
Growth-time and footprint-dependent morphological evolution of square VACNT microstructures. (a) Overview SEM images of the patterned VACNT arrays grown for *t*_0.5_, *t*_1.0_, *t*_1.5_, and *t*_4.0_. In the first overview image, the analyzed 1 nm Fe rows and the qualitative 2 nm Fe rows are indicated, together with the footprint sequence from *L*_12.5_ to *L*_75_. Scale bars: 250 µm. (b) Higher magnification SEM images of representative *L*_75_ structures, which exhibited the most upright morphology across the investigated growth conditions. Scale bars: 50 µm. (c) Higher magnification SEM images of representative deformed structures from the smaller footprint groups. *L*_12.5_ structures are shown for *t*_0.5_ and *t*_1.0_, *L*_25_ structures for *t*_1.5_, and both *L*_25_ and *L*_12.5_ structures for *t*_4.0_ to illustrate the advanced curvature observed at the longest growth condition. Scale bars: 20 µm.

Fig. 2 presents the morphological evolution of the patterned VACNT microstructures as a function of growth time and square catalyst footprint size. The overview SEM images in Fig. 2a show one representative chip from each of the four growth time conditions, *t*_0.5_, *t*_1.0_, *t*_1.5_, and *t*_4.0_, and should therefore not be interpreted as sequential observations of the same structures. Three independent growth experiments were performed for each growth time condition, as described in Section 2.1. Within each image, the footprint size decreases from *L*_75_ at the bottom to *L*_12.5_ at the top, allowing footprint-dependent morphology to be compared under the same growth conditions. The foreground structures grown from 1 nm Fe catalyst patterns were used for all quantitative analyses, whereas the adjacent 2 nm Fe structures were retained only for qualitative comparison, as indicated in Fig. 2a. Higher magnification images of representative *L*_75_ structures are shown in Fig. 2b, while Fig. 2c shows representative deformed structures from the smaller-footprint groups.

At *t*_0.5_, the analyzed structures exhibited a predominantly columnar morphology, although greater apparent base to top offsets were already visible for the smaller footprints. The higher magnification *L*_12.5_ images in Fig. 2c show that this departure from verticality becomes clearly discernible with increasing growth time. At *t*_1.0_, the *L*_12.5_ structures exhibit pronounced lateral deflection, whereas the corresponding *L*_75_ structures in Fig. 2b remain comparatively upright. With further growth, the footprint-dependent differences become increasingly evident. The *L*_75_ structures generally retain a columnar morphology, while *L*_25_ and *L*_12.5_ develop progressively stronger lateral displacement and curvature. The *L*_50_ structures exhibit an intermediate response.

At *t*_4.0_, the morphological contrast is particularly pronounced. The *L*_75_ structures largely preserve their columnar form, whereas the high magnification images of *L*_25_ and *L*_12.5_ in Fig. 2c directly reveal substantial curvature and changes in final orientation. In particular, the strongly curved morphology of the smallest structures illustrates that advanced deformation cannot necessarily be represented as a simple rigid-body inclination. Consequently, the net base to top displacement may not capture the full geometric complexity once pronounced curvature and out of plane redirection develop. Nevertheless, the SEM observations demonstrate a clear increase in the tendency toward loss of verticality with decreasing footprint size and increasing growth time, and therefore with the associated evolution of VACNT height. This behavior is quantified in the following section using the projected relative displacement metric *D*.

### 3.2 Footprint-dependent distribution of projected relative displacement

**Fig. 3:**
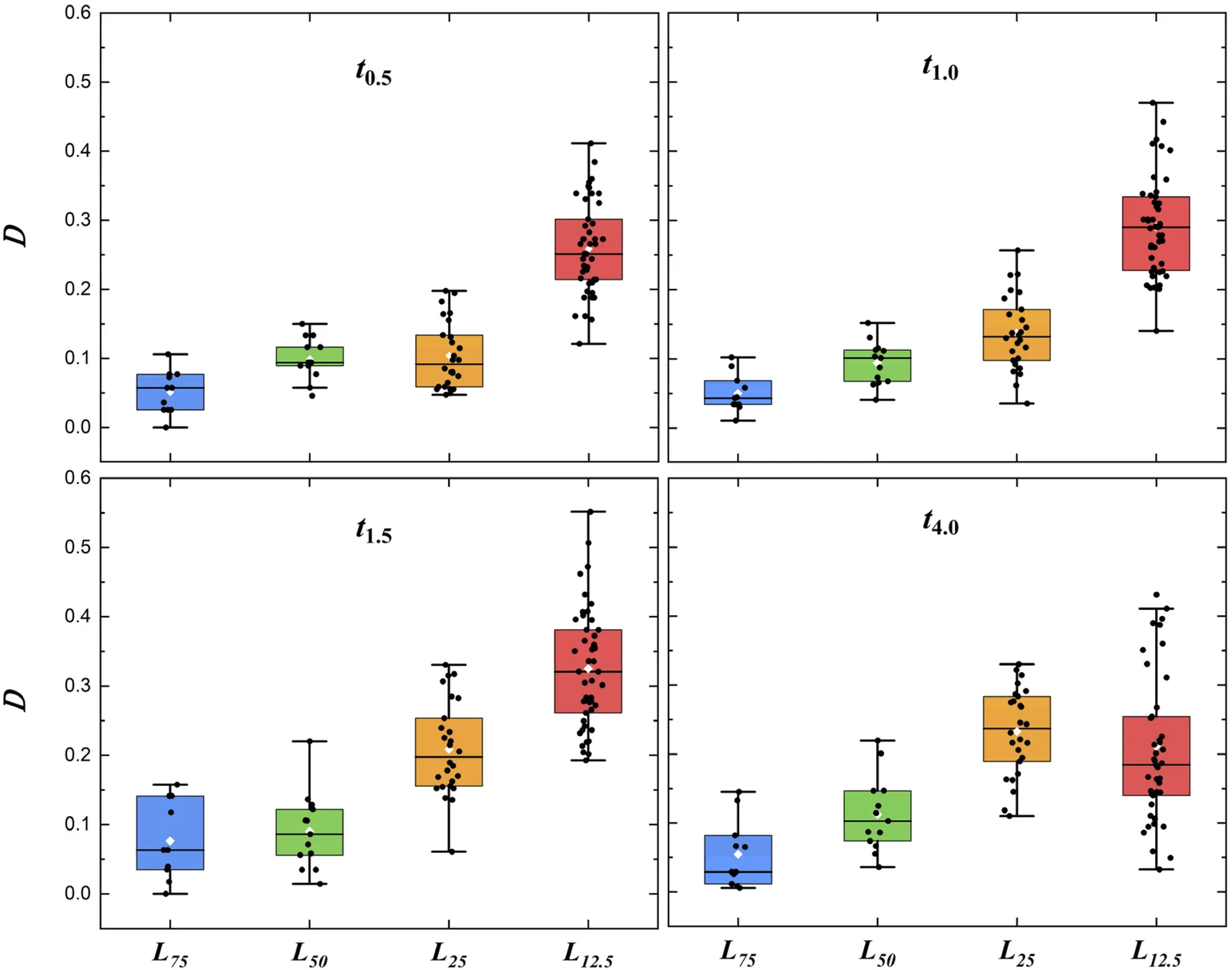
Footprint-dependent distributions of projected relative displacement at fixed growth time. Box plots show the dimensionless projected relative displacement, *D*, for *L*_75_ (blue), *L*_50_ (green), *L*_25_ (orange), and *L*_12.5_ (red) at growth times of *t*_0.5_, *t*_1.0_, *t*_1.5_, and *t*_4.0_. Boxes span the 25th–75th percentiles, horizontal lines indicate the median, whiskers extend to the most extreme values within 1.5× the interquartile range (IQR), and diamond symbols denote the arithmetic mean. Individual black points represent VACNT microstructures and are structural subsamples within the representative chip analyzed for each growth time condition.

For the representative chip datasets shown in Fig. 3, the footprint-dependent variation in VACNT microstructure deformation was quantified using the projected relative displacement metric, *D*, for each individual structure. At all four growth times, the *L*_75_ population exhibited the lowest displacement within the selected reference framework, while decreasing footprint size generally shifted the distributions toward larger *D* values and increased structure to structure variability.

At *t*_0.5_, the median *D* values were approximately 0.057, 0.094, 0.092, and 0.251 for *L*_75_, *L*_50_, *L*_25_, and *L*_12.5_, respectively. Although the intermediate *L*_50_ and *L*_25_ populations did not follow a strictly monotonic order, the *L*_12.5_ structures already exhibited substantially greater deformation. With increasing growth time, the separation between the larger and smaller footprints became more pronounced. At *t*_1.0_, the corresponding median values were 0.043, 0.101, 0.132, and 0.290, while at *t*_1.5_ they were 0.063, 0.086, 0.198, and 0.321. The progressively higher values and broader distributions of *L*_25_ and *L*_12.5_ indicate greater deformation and morphological heterogeneity for the smaller footprints.

At *t*_4.0_, the median *D* values were 0.029, 0.103, 0.237, and 0.185 for *L*_75_, *L*_50_, *L*_25_, and *L*_12.5_, respectively. The *L*_25_ population therefore exceeded *L*_12.5_, but this reversal does not indicate recovery of vertical stability in the smallest structures. Rather, pronounced curvature, out of plane redirection, and possible interactions between neighboring structures may reduce the extent to which the net base to top displacement captures the full deformation. Overall, *L*_75_ remained in a low deformation regime, *L*_50_ showed an intermediate response, *L*_25_ exhibited the strongest growth time-dependent increase, and *L*_12.5_ entered a high deformation regime from the earliest growth condition.

The detailed distributions in Fig. 3 correspond to one representative chip from each growth time condition. To evaluate synthesis to synthesis reproducibility, the same *D* analysis was performed independently for two additional chips at each growth time, giving three independent growth experiments per condition. The chip-level median *D* values reproduced the same overall footprint-dependent behavior across the three independent syntheses (Fig. S1). In particular, *L*_75_ consistently remained the lowest-displacement population, the pronounced increase in *D* toward the smaller footprints was reproduced at *t*_1.0_ and *t*_1.5_, and the *L*_25_ > *L*_12.5_ crossover observed at *t*_4.0_ was reproduced in all three independently grown chips. Thus, the footprint and growth-dependent trends observed in the detailed distributions are not specific to a single synthesis.

### 3.3 Transition from low to advanced deformation regimes

**Fig. 4:**
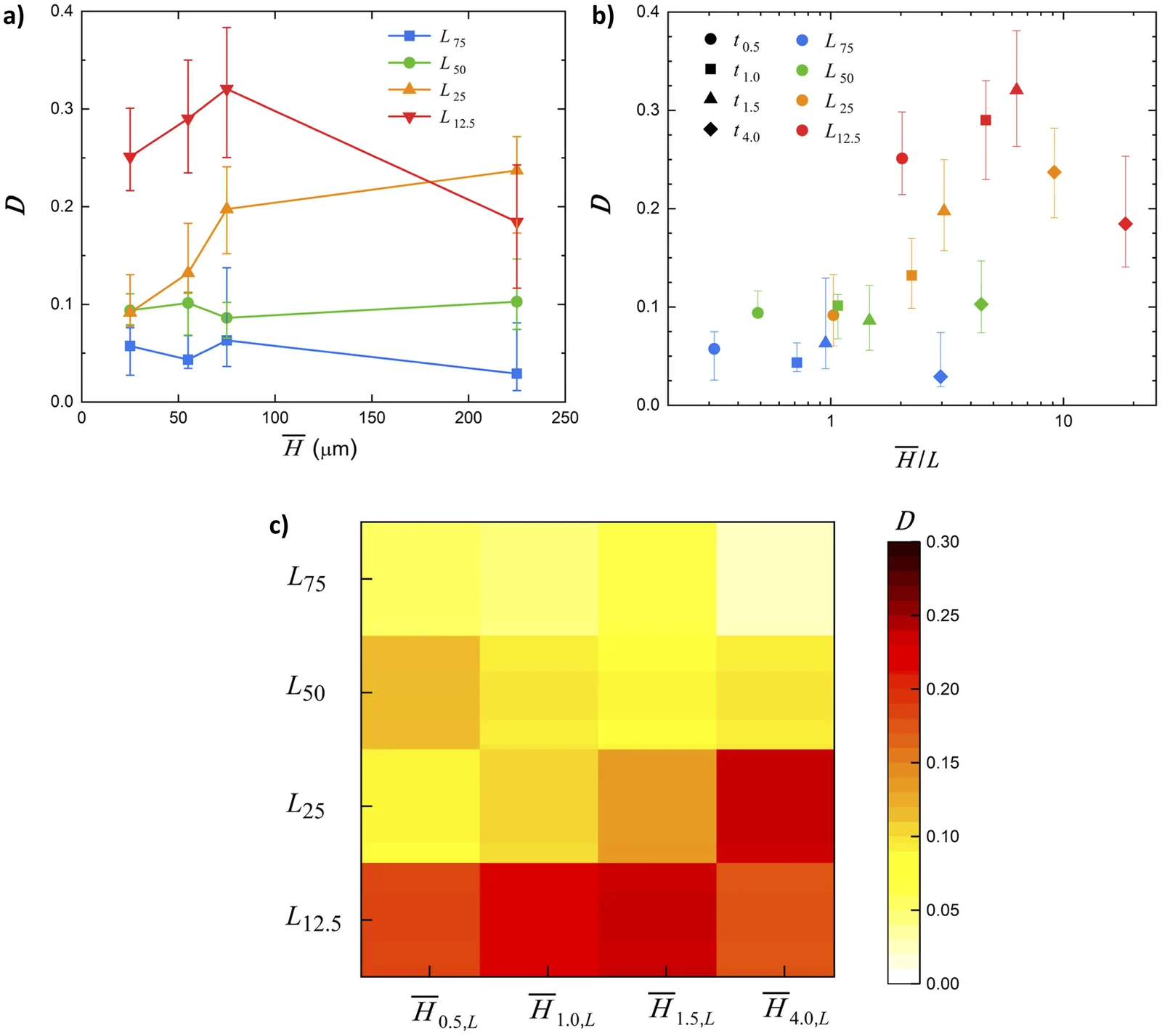
Combined effects of VACNT height and catalyst footprint size on projected microstructure deformation. (a) Evolution of the median projected relative displacement, *D*, with the footprint-specific mean VACNT height, *H̄*_*t*,*L*_, for *L*_75_, *L*_50_, *L*_25_, and *L*_12.5_. Error bars span the 25th–75th percentiles. Lines are included only as guides to the eye. The plotted median values correspond to the representative chip datasets used in Fig. 3 and are not sequential measurements of the same structures. (b) Median *D* plotted against the geometric aspect ratio *H̄*_*t*,*L*_ on a logarithmic horizontal axis. Colors identify footprint size and symbol shapes represent the growth time conditions *t*_0.5_, *t*_1.0_, *t*_1.5_, and *t*_4.0_. Error bars indicate the interquartile range of individual structure *D* values within the representative chip. (c) Height-footprint map of median *D*, with footprint size arranged from *L*_75_ to *L*_12.5_ and columns corresponding to the footprint-specific mean heights obtained at *t*_0.5_, *t*_1.0_, *t*_1.5_, and *t*_4.0_.

The combined influence of VACNT height and catalyst footprint size is summarized in Fig. 4. Rather than following a single monotonic relationship, the four footprint populations exhibited distinct height-dependent deformation trajectories. The *L*_75_ structures remained within a consistently low displacement regime over the investigated range, with median *D* values between approximately 0.03 and 0.06. The *L*_50_ structures showed an intermediate response, with median values remaining close to 0.09–0.10 and only a limited dependence on VACNT height. These results indicate that the two largest footprints retained comparatively low and weakly height-dependent projected deformation.

A markedly different evolution was observed for the smaller footprints. For *L*_25_, the median projected relative displacement increased progressively from 0.092 at *t*_0.5_ to 0.132, 0.198, and 0.237 at *t*_1.0_, *t*_1.5_, *t*_4.0_, respectively. This pronounced increase with the corresponding footprint-specific mean height identifies *L*_25_ as a transition footprint whose deformation evolves from a relatively moderate response toward the advanced deformation regime. By contrast, the *L*_12.5_ structures already exhibited a high median displacement of 0.251 at *t*_0.5_, which further increased to 0.290 and 0.321 at *t*_1.0_ and *t*_1.5_, respectively. At *t*_4.0_, however, the median decreased to 0.185, and the *L*_25_ population exhibited the larger projected displacement. This crossover should not be interpreted as recovery of vertical stability in the smallest structures. At large heights and small footprints, deformation is no longer necessarily represented by a simple single direction inclination. Strong curvature, out of plane redirection, and possible interactions between neighboring structures can alter the final position of the upper face without proportionally increasing the projected base to top displacement. Consequently, *D* remains a useful measure of overall projected deviation but may underestimate the full geometric complexity of structures that have entered an advanced deformation regime.

Plotting the median *D* values against the geometric aspect ratio *H̄*/*L* revealed a general tendency toward larger deformation at higher aspect ratios, but the data did not collapse onto a unique master relationship (Fig. 4b). In particular, structures with nearly identical aspect ratios could exhibit substantially different deformation levels depending on their absolute footprint and height. For example, the *L*_25_ structure at *t*_1.5_ and the *L*_75_ structure at *t*_4.0_ have very similar aspect ratios, *H̄*/*L* = 3.067 and 2.964, respectively, yet their median *D* values differ markedly, 0.198 and 0.029. Thus, *H̄*/*L* captures an important geometric contribution to deformation susceptibility but is not sufficient by itself to describe the complete as-grown response. The comparison also indicates that absolute footprint size and the growth history associated with reaching a given height contribute to the observed morphology.

The height-footprint map in Fig. 4c further illustrates the emergence of distinct deformation regimes. The *L*_75_ row remains within the lowest *D* range across all growth conditions, whereas *L*_50_ occupies an intermediate and comparatively uniform region. The *L*_25_ structures show a progressive shift toward higher deformation as their mean height increases. In contrast, *L*_12.5_ occupies the high deformation region from the earliest growth condition and reaches its largest median *D* at *t*_1.5_, followed by a reduction at *t*_4.0_. The latter behavior is consistent with a regime in which further morphological evolution is not necessarily reflected by an increasing net projected base to top displacement. The results support three descriptive response regimes: (i) a low deformation regime associated primarily with *L*_75_, (ii) an intermediate regime represented by *L*_50_, with *L*_25_ undergoing a pronounced height-dependent transition through this regime, and (iii) an early-onset advanced deformation regime associated with *L*_12.5_. These regimes are empirical descriptions based on the measured projected displacement and should not be interpreted as mechanically defined stability boundaries. The absence of a universal *D*–*H̄*/*L* relationship motivates comparison with an idealized elastic stability baseline, while the substantially different experimental responses observed at nearly identical *H̄*/*L* demonstrate that additional absolute size and growth-dependent effects are not captured by aspect ratio alone.

### 3.4 Geometry-dependent stability of patterned VACNT microstructures

**Fig. 5:**
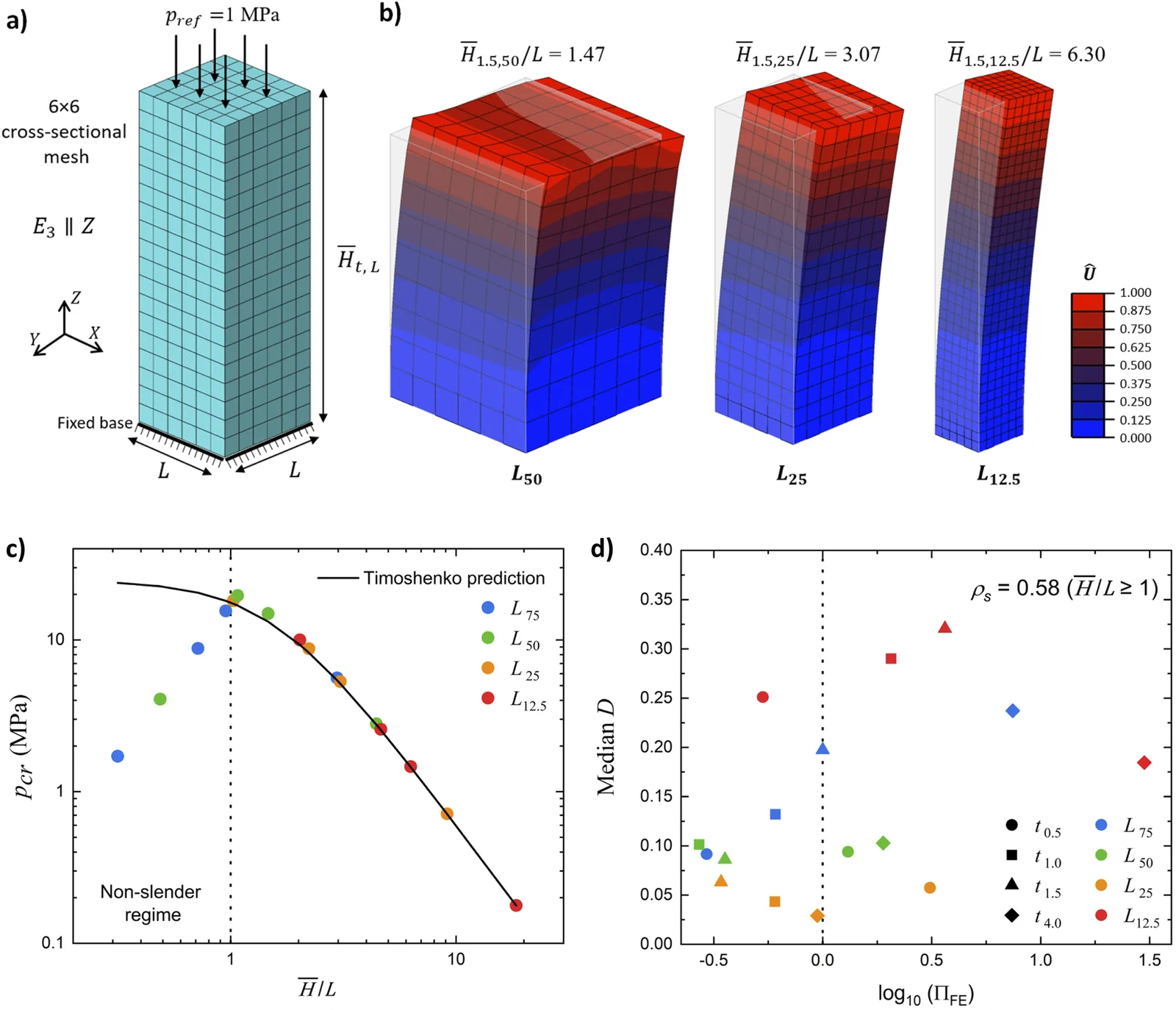
Finite element analysis of geometry-dependent stability in patterned VACNT microstructures. (a) Orthotropic finite element representation of a square VACNT microstructure with footprint *L* × *L* and footprint-specific mean height *H̄*_*t*,*L*_. A 6 × 6 cross-sectional mesh was used following mesh convergence verification. (b) Representative first global eigenvalue-buckling modes for the *t*_1.5_ growth condition for *L*_50_, *L*_25_, and *L*_12.5_, corresponding to *H̄*/*L* = 1.47, 3.07, and 6.30, respectively. Transparent outlines indicate the undeformed geometries, and the color scale represents the normalized eigenmode displacement amplitude, *Û*, calculated separately for each model. (c) FE critical pressure, *p*_cr,FE_, as a function of *H̄*/*L*. Symbol colors identify footprint size, and the solid curve represents the shear-corrected Timoshenko prediction. The vertical dotted line at *H̄*/*L* = 1 separates the non-slender region from the beam-column regime. (d) Experimental median projected relative displacement, *D*, as a function of the FE-based susceptibility index, *Π*_FE_. Symbol colors indicate footprint size and symbol shapes indicate growth time. The vertical dotted line corresponds to *Π*_FE_ = 1; the Spearman coefficient shown corresponds to the 12 conditions satisfying *H̄*/*L* ≥ 1.

The eigenvalue-buckling analysis revealed a strong dependence of the mechanical stability of the patterned VACNT microstructures on geometry (Fig. 5). The representative mode shapes for the *t*_1.5_ growth condition illustrate the effect of decreasing footprint size (Fig. 5b). For *L*_50_, *L*_25_, and *L*_12.5_, the corresponding aspect ratios were *H̄*/*L* = 1.47, 3.07, and 6.30, respectively. All three geometries exhibited a global lateral bending mode, while the increasingly slender structures showed a progressively more pronounced column-like response. The color contours represent the normalized eigenmode displacement amplitude,
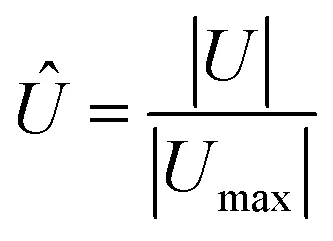
calculated separately for each geometry. Accordingly, the contours describe the spatial distribution of the eigenmode rather than the physical displacement magnitude at the critical load, and the amplitudes should not be compared directly between geometries.

The calculated FE critical pressure, *p*_cr,FE_, decreased systematically with increasing *H̄*/*L* (Fig. 5c), demonstrating a strong reduction in elastic stability as the structures became more slender. For geometries with *H̄*/*L* ≥ 1, the FE results were compared with the shear-corrected Timoshenko prediction,
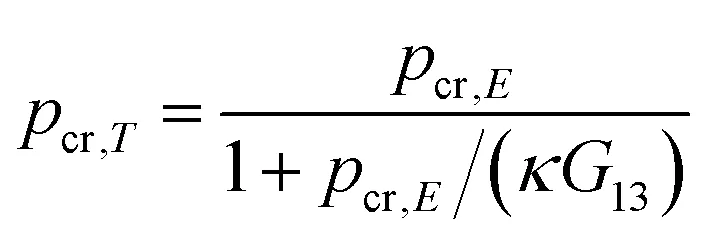
where
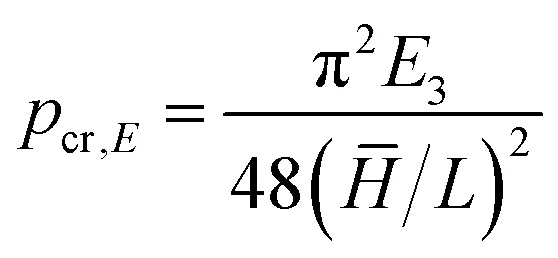
is the corresponding Euler estimate for a fixed-free square column and *κ* = 5/6 is the shear correction factor. The agreement between the FE and Timoshenko results improved with increasing *H̄*/*L*, indicating that the response approaches a global flexural regime as slenderness increases. In contrast, the four geometries with *H̄*/*L* < 1 deviated substantially from the beam-column prediction. These short and laterally broad structures lie outside the classical slender column regime, where transverse shear, three-dimensional deformation, and end constraint effects become comparatively more important.

To relate the calculated stability to the experimentally observed loss of verticality, a dimensionless FE-based susceptibility index was defined as
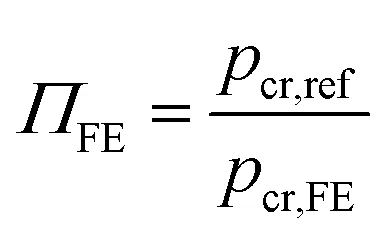
where *p*_cr,ref_ was taken as the FE critical pressure of the *L*_25_–*t*_1.5_ condition. Thus, *Π*_FE_ = 1 for the selected reference geometry, while *Π*_FE_ < 1 indicates greater calculated stability and *Π*_FE_ > 1 indicates greater susceptibility to instability. Because all FE models share the same orthotropic material properties, square cross-sectional shape, and boundary conditions, *p*_cr,FE_, and consequently *Π*_FE_, are governed predominantly by *H̄*/*L*. The index is therefore interpreted as a mechanically motivated slenderness-based susceptibility measure rather than as an independent predictor of the experimental deformation.

The experimental median *D* generally increased with log_10_(*Π*_FE_) within the beam-column regime (Fig. 5d). A descriptive Spearman rank coefficient of *ρ*_s_ = 0.58 was obtained for the 12 conditions satisfying *H̄*/*L* ≥ 1, indicating a moderate positive correspondence between the experimentally observed deformation and the eigenvalue-based susceptibility. Structures predicted to be less stable therefore tended, in general, to exhibit greater as-grown projected deformation. However, the substantial scatter in Fig. 5d demonstrates that the relationship is not sufficiently unique for *Π*_FE_ to be regarded as a direct predictor of *D*.

The limitations of the idealized stability description become particularly evident when geometries with nearly identical aspect ratios are compared. The *L*_25_–*t*_1.5_ and *L*_75_–*t*_4.0_ conditions have *H̄*/*L* = 3.067 and 2.964, respectively, and correspondingly similar FE critical pressures of 5.3148 and 5.6197 MPa. Nevertheless, their experimental median *D* values are markedly different, 0.198 and 0.029, respectively. Thus, nearly equivalent idealized elastic susceptibility can correspond to substantially different as-grown morphologies. This observation is consistent with the experimental results in Fig. 4, where the deformation data did not collapse onto a universal *H̄*/*L* relationship. The eigenvalue analysis captures the geometry-dependent susceptibility to the onset of mechanical instability, whereas the final experimental morphology additionally reflects growth history, structural heterogeneity, post buckling evolution, out of plane redirection, and possible interactions between neighboring VACNT microstructures. The model should therefore be interpreted as a comparative mechanical stability baseline rather than as a direct reconstruction of the final deformation.

The material parameter sensitivity analysis further showed that the influence of the assumed orthotropic stiffness contrast depends strongly on slenderness (Fig. S2). Near *H̄*/*L* ≈ 1, changing *E*_3_/*G*_13_ from the baseline value of 10 to 20 or 5 altered *p*_cr,FE_ by approximately −50% and +64%, respectively. This sensitivity decreased substantially with increasing aspect ratio, with deviations from the baseline falling below approximately 3% at *H̄*/*L* ≈ 9 and below 1% at *H̄*/*L* ≈ 18. Thus, within the idealized FE model, the stability of highly slender structures is controlled predominantly by global flexural geometry, whereas the predicted critical pressure of shorter structures remains considerably more sensitive to the assumed transverse and shear properties.

## 4 Conclusion

This study quantified the geometry-dependent loss of verticality in lithographically patterned VACNT microstructures across four catalyst footprint sizes and four growth time conditions. The largest footprint, *L*_75_, consistently exhibited the lowest projected deformation throughout the investigated range, whereas *L*_50_ showed an intermediate response. The *L*_25_ structures exhibited the strongest growth-dependent transition toward higher deformation, while *L*_12.5_ entered a highly deformed regime from the earliest growth condition. The experimental data showed a general increase in deformation with *H̄*/*L*, but did not collapse onto a universal relationship, demonstrating that footprint size and VACNT height cannot be treated as fully interchangeable geometric parameters.

The orthotropic FE analysis provided an idealized mechanical baseline for interpreting this trend. The critical pressure decreased systematically with increasing *H̄*/*L*, and the numerical results approached the shear-corrected Timoshenko prediction in the beam-column regime. The FE-based susceptibility index showed a moderate positive correspondence with the experimental deformation (*ρ*_s_ = 0.58 for *H̄*/*L* ≥ 1), supporting geometry-dependent elastic instability as an important contributor to the loss of verticality. However, the remaining scatter and the markedly different deformation observed for geometries with similar aspect ratios show that the final as-grown morphology is not determined by slenderness alone. The proposed framework therefore provides a comparative framework for identifying footprint–height combinations more likely to preserve the intended vertical architecture of patterned VACNT microstructures.

## Author contributions

Ozlem Simsek: investigation, formal analysis, data curation. O. Tolga Gul: methodology, formal analysis, investigation, supervision, conceptualization, software, data curation, visualization, validation, writing – original draft, review and editing.

## Conflicts of interest

The authors declare that they have no known competing financial interests or personal relationships that could have appeared to influence the work reported in this paper.

## Supplementary Material

RA-OLF-D6RA07679C-s001

RA-OLF-D6RA07679C-s002

## Data Availability

The data supporting this article have been included as part of the supplementary information (SI). Supplementary information is available. See DOI: https://doi.org/10.1039/d6ra07679c.
