# Supplementary material for: When vertically aligned carbon nanotube microstructures lose verticality: experimental and computational insights into geometry-dependent stability
Source: RSC Adv. 2026 Sep 15;16(44):55231–42. doi: 10.1039/d6ra07679c (PMC13575859; doi:10.1039/d6ra07679c)
Supplement: Supplementary file 1 [file RA-016-D6RA07679C-s001.pdf]

Supplementary Information (SI) for RSC Advances.
This journal is © The Royal Society of Chemistry 2026

Supplementary Information

When vertically aligned carbon nanotube microstructures lose verticality: experimental and computational insights into geometry-dependent stability

Ozlem Simsek[1], O. Tolga Gul[1]*

Ozlem Simsek: Physics Department, Polatlı Faculty of Science and Letters, Ankara Hacı Bayram Veli University, Ankara 06900, Türkiye

O. Tolga Gul: Physics Department, Polatlı Faculty of Science and Letters, Ankara Hacı Bayram Veli University, Ankara 06900, Türkiye; https://orcid.org/0000-0003-2033-1205

E-mail: tolga.gul@hbv.edu.tr

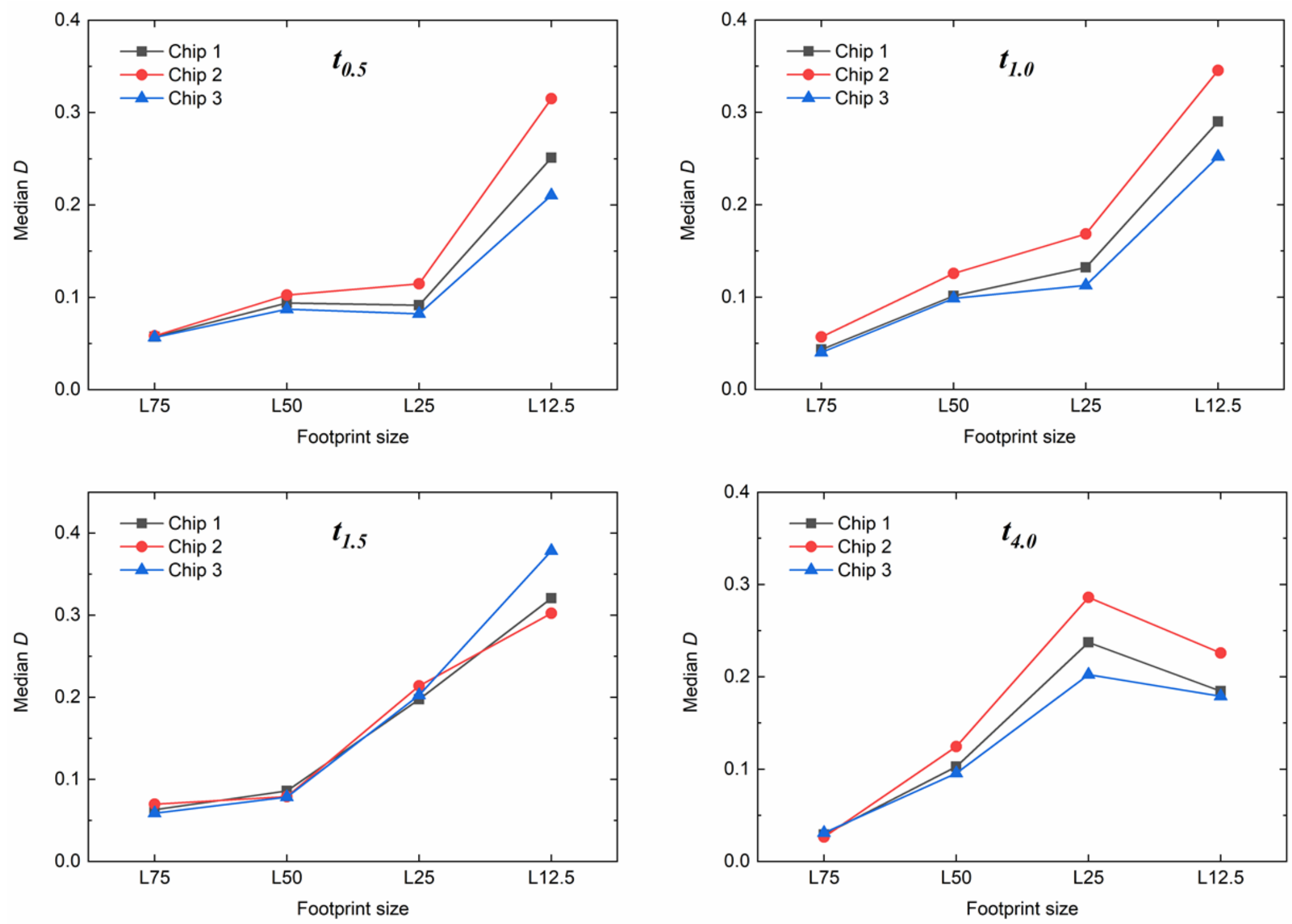

Figure S1. Reproducibility of footprint-dependent projected deformation across independent growth experiments. Chip-level median projected relative displacement, D, for L_{75}, L_{50}, L_{25}, and $L_{12.5}$ structures obtained from three independently grown chips at **(a)** $t_{0.5}$, **(b)** $t_{1.0}$, **(c)** $t_{1.5}$, and **(d)** $t_{4.0}$,. Each line represents an independently grown chip. For each chip, D was calculated independently using the median L_{75} response of the same chip as the internal reference. Individual VACNT structures within each chip were treated as structural subsamples, whereas the three independently grown chips represent synthesis replicates. Lines are included as guides to the eye.

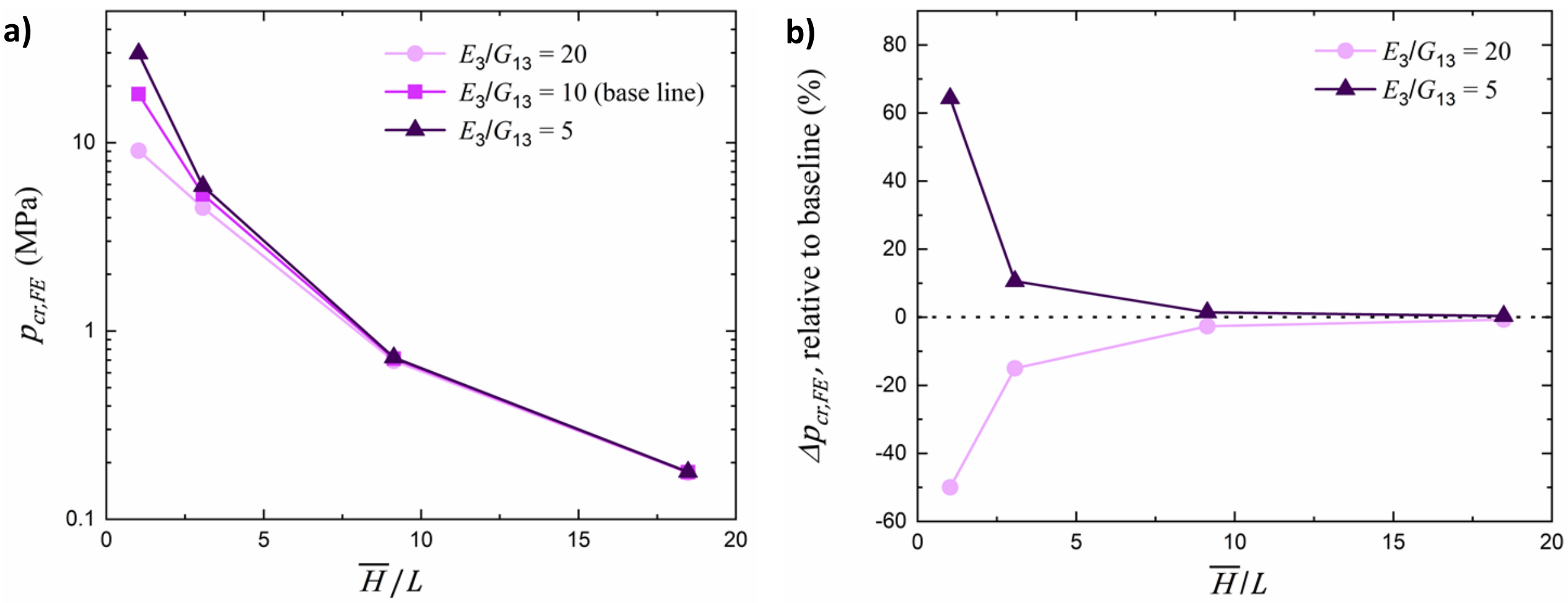

Figure S2. Sensitivity of the FE critical pressure to the assumed orthotropic stiffness contrast. Four representative geometries were selected from the L_t matrix: $L_{25}\_t_{0.5}$, $L_{25}\_t_{1.5}$, $L_{25}\_t_{4.0}$, and $L_{12.5}\_t_{4.0}$, corresponding to $\overline{H}/L$≈1.03, 3.07, 9.13, and 18.48, respectively. The longitudinal modulus was maintained at E_3=297 MPa, while the transverse and shear moduli were varied to produce E_3/G_{13} ratios of 20, 10 (baseline), and 5. **(a)** FE critical pressure, $p_{cr,FE}$, as a function of $\overline{H}/L$. (b) Percentage change in $p_{cr,FE}$ relative to the baseline property set (E_3/G_{13}=10). The influence of the assumed transverse and shear stiffnesses decreases markedly with increasing $\overline{H}/L$. Lines are included as guides to the eye.

Table S1. Material properties, discretization, loading, and solver settings used in the eigenvalue-buckling analyses.

**Parameter**	**Value**
$E_1 = E_2$	29.7 MPa
E_3	297 MPa
G_{12}	14.85 MPa
$G_{13} = G_{23}$	29.7 MPa
ν_{12},ν_{13},ν_{23}	0
Element type	C3D8I
Cross-sectional mesh	6×6
Base condition	Fully fixed
Reference pressure	1 MPa
Eigensolver	Subspace
Requested eigenmodes	8
Material direction	$E_3 \parallel Z$

Table S2. Mesh convergence analysis for two representative real height VACNT geometries.

Growth time (min)	L (μm)	$\overline{H}$ (μm)	$\overline{H}/L$ (-)	Cross-sectional mesh	Elements	$p_{cr,FE}$ (MPa)	Difference from 8×8 (%)*
1.5	25	76.67	3.067	4×4	208	5.3557	1.070
1.5	25	76.67	3.067	6×6	684	5.3148	0.298
1.5	25	76.67	3.067	8×8	1600	5.2990	0
4.0	12.5	231.00	18.480	4×4	1184	0.17771	0.0281
4.0	12.5	231.00	18.480	6×6	3996	0.17767	0.0056
4.0	12.5	231.00	18.480	8×8	9472	0.17766	0

* The percentage difference was calculated relative to the corresponding 8 ×8 cross-sectional mesh result. The difference between the selected 6×6 mesh and the 8×8 mesh remained below 0.3% for both representative geometries.

Table S3. Experimental deformation metrics and eigenvalue-buckling results for the complete L–t matrix.

Growth time (min)	L (μm)	$\overline{H}$ (μm)	$\overline{H}/L$ (-)	n* (-)	Median (D) (-)	IQR**	$p_{cr,FE}$ (MPa)	$p_{cr,T}$ (MPa)	$p_{cr,FE}/p_{cr}$ (-)	Π_{FE}*** (-)
0.5	75	23.66	0.316	11	0.057	0.049	1.7119	23.789	0.072	3.105
0.5	50	24.33	0.487	13	0.094	0.027	4.0719	22.582	0.180	1.305
0.5	25	25.66	1.027	26	0.092	0.073	18.122	17.342	1.045	0.293
0.5	12.5	25.33	2.027	47	0.251	0.084	10.002	9.288	1.077	0.531
1.0	75	53.66	0.716	11	0.043	0.029	8.8029	20.497	0.429	0.604
1.0	50	53.66	1.073	13	0.101	0.045	19.609	16.872	1.162	0.271
1.0	25	55.66	2.227	26	0.132	0.071	8.753	8.224	1.064	0.607
1.0	12.5	58.00	4.640	47	0.290	0.100	2.5792	2.545	1.014	2.061
1.5	75	71.33	0.951	11	0.063	0.092	15.552	18.110	0.859	0.342
1.5	50	73.33	1.467	13	0.086	0.066	14.935	13.222	1.130	0.356
1.5	25	76.66	3.067	26	0.198	0.093	5.3148	5.144	1.033	1.000
1.5	12.5	78.66	6.293	47	0.321	0.118	1.4617	1.451	1.007	3.636
4.0	75	222.33	2.964	11	0.029	0.055	5.6197	5.426	1.036	0.946
4.0	50	221.66	4.433	13	0.103	0.073	2.8017	2.760	1.015	1.897
4.0	25	228.33	9.133	26	0.237	0.091	0.71334	0.711	1.003	7.451
4.0	12.5	231.00	18.480	47	0.185	0.112	0.17767	0.178	1.000	29.914

* The number of structures for each footprint size within each chip.

** Interquartile Range

*** $\Pi_{FE} = \dfrac{p_{cr,ref}}{p_{cr,FE}}$
